# LightTrack-ReID: A lightweight and occlusion-robust framework for multi-object tracking

**DOI:** 10.1371/journal.pone.0342246

**Published:** 2026-03-25

**Authors:** Said Baz Jahfar Khan, Peng Zhang, Mian Muhammad Kamal, Abdul Khader Jilani Saudagar

**Affiliations:** 1 School of Software Engineering, Northwestern Polytechnical University, Xi’an, Shaanxi, China; 2 Ningbo Institute of Northwestern Polytechnical University, Beilun, Ningbo, China; 3 School of Computer Science, Northwestern Polytechnical University, Xi’an, Shaanxi, China; 4 School of Electronic and Communication Engineering, Quanzhou University of Information Engineering, Quanzhou, Fujian, China; 5 Information Systems Department, College of Computer and Information Sciences, Imam Mohammad Ibn Saud Islamic University (IMSIU), Riyadh, Saudi Arabia; Chongqing Normal University, CHINA

## Abstract

This paper presents LightTrack-ReID, an advanced, lightweight, and occlusion-resistant framework for MOT, designed for real-time performance in resource-limited environments. The framework includes a Lightweight Appearance Encoder (LAE) using MobileNetV3-Small, Transformer-Based Similarity Scoring (TBSS), Context Memory for Occlusion Handling (CMOH), and Adaptive Similarity Weighting (ASW) to enhance tracklet association in situations of heavy occlusion. These components offer compact 32-dimensional ReID features, adaptive similarity metrics, and continuous tracking within an efficient single-stage detection-to-tracklet association system. The proposed similarity and association model operates at approximately 0.6 GFLOPs per frame (LAE approximately 0.5 GFLOPs + TBSS approximately 0.1 GFLOPs). When integrated with the YOLOX-S detector, which remains the dominant computation, the full pipeline maintains approximately 30 FPS real-time performance on a GTX1080 GPU. It demonstrates robust performance on the MOT17 and MOT20 benchmarks, achieving Higher Order Tracking Accuracy(HOTA) scores of 66.92 and 66.6 and IDentity F1 score(IDF1) scores of 82.52 and 82.2, respectively, while significantly reducing identity switches. These results confirm its strength and appropriateness for use in real-world applications.

## 1 Introduction

Multi-Object Tracking (MOT) is a core task in computer vision that focuses on detecting objects in video frames and associating them over time to establish coherent trajectories [1,2]. The main objective is to assign and sustain distinct IDs for objects over time, offering effective tracking despite challenges such as occlusions, abrupt movements, and complex interactions [2,3]. MOT encourages important applications, such as autonomous driving safety [4], enhanced surveillance systems [5], ecological monitoring via animal tracking [6], enhanced human-robot cooperation [7], and effective video and sports analytics [8]. Effective multi-object tracking requires accurate object detection in each frame and reliable association with existing tracklets, including assigning new identities to unmatched objects and terminating trajectories of objects leaving the scene [2,3,9,10] MOT methods are categorized into offline and online models. Offline methods process entire video sequences to improve accuracy, whereas online techniques, essential to real-time applications, process frames sequentially [11–13]. Online MOT predominantly adopts Tracking-by-Detection (TBD), which separates detection from association, or Joint Detection and Tracking (JDT), which combines both for improved computing efficiency [1,3,14]. Recent developments in TBD, propelled by methods such as YOLO, have significantly improved detection speed and accuracy, making it very suitable for real-time applications [15]. The association in TBD often employs the Hungarian method [7], which uses a cost matrix based on similarity measures such as Intersection over Union (IoU), Mahalanobis distance [16], and appearance-based cosine similarity [11,17]. Confidence-based filtering decreases false positives but may inadvertently eliminate legitimate detections. [18].

Occlusion is an important challenge in multi-object tracking, which commonly results in detection failures and trajectory fragmentation. Re-Identification (ReID) methods address this issue by reconnecting inactive tracklets between frames [19]. Methods such as DeepSort [20] use standalone ReID models, but JDE methods, exemplified by FairMOT [21], incorporate ReID into the detection framework. Recent developments leverage advanced deep learning techniques, such as attention mechanisms [22,23], graph neural networks [24], and hierarchical feature extraction [25], to model motion dynamics and contextual signals for occlusion-resistant tracking.

**Fig 1:**
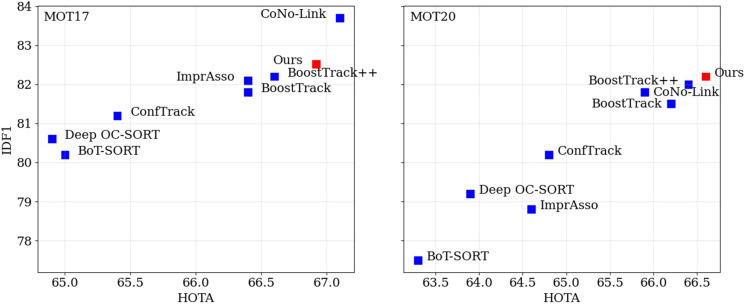
HOTA and IDF1 metric results on MOT17 (left) and MOT20 (right) test sets.

In this paper, we propose LightTrack-ReID, an innovative and highly robust multi-object tracking (MOT) framework that achieves superior effectiveness, as demonstrated by the comparative performance compared to the present state-of-the-art method illustrated in Figure 1. [2,3,9,10].

**Fig 2:**
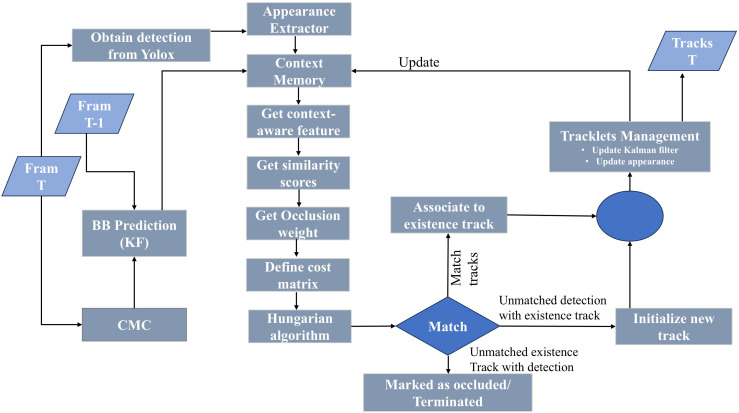
Overview of the proposed model architecture.

The proposed framework integrates several novel components, including a Lightweight Appearance Encoder (LAE), Transformer-Based Similarity Scoring (TBSS), Context Memory for Occlusion Handling (CMOH), and Adaptive Similarity Weighting (ASW), as shown in Figure 2.

The method also employs a single-stage detection-to-tracklet association structure that incorporates confidence similarity boosting and enhances detection confidence specifically for low-confidence tracking. Our key contributions are as follows:

- A Lightweight Appearance Encoder (LAE) utilizing MobileNetV3-Small, providing compact 32-dimensional ReID features for expedited inference;
- Transformer-Based Similarity Scoring (TBSS), integrating a single-layer transformer for strong, low-overhead association;
- Context Memory for Occlusion Handling (CMOH), which keeps track continuity during occlusions by using recent appearance embeddings;
- Adaptive Similarity Weighting (ASW), which easily changes the impact of IoU and appearance similarity in accordance with occlusion density.

**Fig 3:**
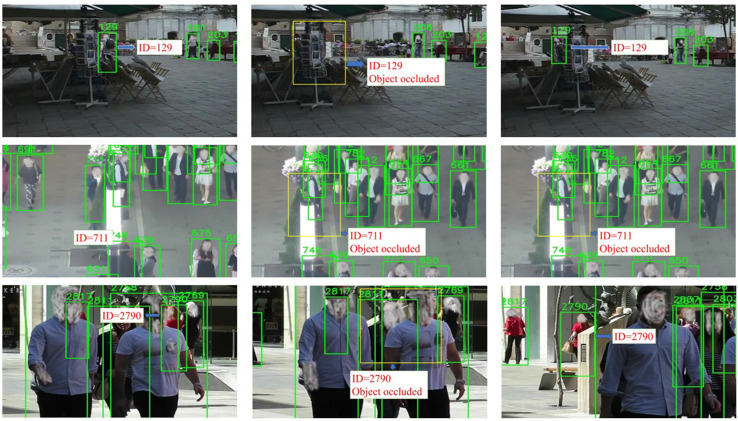
Each row shows one sequence: the object before occlusion, during occlusion, and after reappearing with the same ID.

Figure 3 presents bounding boxes and corresponding objects as produced by our model on the MOT17 dataset. The figure illustrates images grouped in three rows (one for each sequence), with each row showing the object before occlusion, during occlusion, and after its reappearance, maintaining the same ID accurately recorded.

LightTrack-ReID shows outstanding outcomes on the MOT17 and MOT20 benchmarks, achieving HOTA scores of 66.92 and 66.6 and IDF1 scores of 82.52 and 82.2, respectively—surpassing multiple existing methodologies while maintaining real-time efficiency.

The main contribution of this research lies in the design of an efficiency-oriented tracking system rather than proposing an entirely new framework. LightTrack-ReID proposes a synergistic integration of a compact appearance encoder, a single-layer association transformer, contextual memory, and occlusion-aware similarity regulation. This integration concurrently provides real-time efficiency of 0.6 GFLOPs and robustness under significant occlusion, a feat not achieved by current high-complexity or limited-feature methodologies.

Note: Unless otherwise specified, the GFLOPs values, reported in this paper in this paper (e.g., 0.6 GFLOPs) correspond solely to the computational expense of our association/similarity component (LAE + TBSS + lightweight context modules). The comprehensive FPS results encompass the whole detection and association pipeline. The computation of the detector (YOLOX) represents the primary computational burden; enhancements in our association module have minimal impact on the overall GFLOPs.

## 2 Related work

Recent advancements in multi-object tracking (MOT) have mostly focused on improving robustness in crowded environments and computing efficiency. We examine relevant research in three important areas: (1) Tracking Frameworks, (2) Occlusion Handling, and (3) Re-Identification, focusing on the gaps that our method rectifies.

### 2.1 Tracking framework

Online tracking often refers to a tracking-by-detection framework, wherein objects are detected in each frame and linked to existing tracklets [1,3,14]. Kalman filtering [26] is widely used for motion prediction, whereas the Hungarian method [27] is usually used to address the data association problem. A cost matrix using Intersection over Union (IoU) or a mix of IoU [16] and appearance features is used to associate detections with existing tracks [28]. To address occlusions and crowded settings, multiple models include appearance similarity with motion cues. To reduce false positives and ghost tracks, various methods exclude low-confidence detections and associate only with high-confidence tracklets. Recent studies indicate that incorporating low-confidence detections associated with a second stage could recover missed objects and improve recall [29,30]. Multiple studies [31–33] have proposed multi-stage association frameworks that highlight more recently updated or higher-confidence tracklets during the first stages. Although successful, these multi-stage techniques may cause more identity switches due to inconsistent associations between stages. On the other hand, BoostTrack [18] and BoostTrack++ [17] use a single-stage association method. They use confidence boosting, Mahalanobis distance, and appearance similarities, such as visual embeddings and shape similarity, to achieve robust association. BoostTrack++ improves confidence modeling, generating superior outcomes.

### 2.2 Occlusion handling

Occlusion presents a continual challenge in multi-object tracking (MOT), often disrupting detections and fragmenting trajectories. Researchers have developed various methods to maintain track continuity in partial or complete occlusions. TrackFormer [22] uses a transformer-based attention mechanism to represent contextual and spatial relationships between objects. It collects long-range relationships that enhance occlusion resilience; however, it suffers from high computational demands because of huge data requirements. MeMOT [34] uses a transformer-based architecture, including memory encoding for simultaneous detection and data association. It is highly effective in crowded environments with recurrent occlusions. SMILEtrack [14] uses a Siamese Learning Module and Patch Self-Attention to improve feature matching in situations of occlusion. Nonetheless, its fixed similarity metric limits adaptability to varying occlusion levels, potentially reducing association accuracy over time. Occlusion-Aware Attention (OAA) [35] uses a self-supervised mask loss to reduce occluded background areas, therefore focusing the model’s attention on visible features. Although it improves robustness in dense environments, its detection-centric methodology neglects motion continuity, leading to identity switches. DetTrack [36] addresses full occlusion by combining spatio-temporal information with motion cues. It predicts object positions based on motion in missing detections, ensuring continuity without the necessity for explicit visual input. MSPNet [37] (Motion-guided and Occlusion-aware Multi-Object Tracking) integrates motion-guided aggregation with an occlusion-aware attention mechanism. Its hierarchical spatial association considerably improves performance in situations with frequent occlusion and camera movements. PSMOT [38] uses position-sensitive pooling to maintain spatial context within feature maps, boosting robustness against occlusion and lower identity changes in noisy environments.

### 2.3 Re-identification

Re-identification (Re-ID) ensures stable identity tracking across frames, especially after occlusion or missed detections. It uses visual, motion, or combined cues to accurately re-associate missing tracklets. FairMOT [21] integrates Re-ID directly into its joint detection and tracking (JDT) framework, producing embeddings simultaneously with detections, allowing real-time identity association. BoT-SORT [28] adopts a deep CNN-based model, such as OSNet, to extract distinctive appearance features, providing more resilience in occluded and crowded environments. BoostTrack [18] combines motion-based prediction with a simplified appearance model (e.g., SHIP) for hybrid association. It achieves outstanding efficiency without significant feature extraction, but it can fail when appearance or motion features lack distinctiveness. MOTFR [39] uses a Feature Purification Module (FPM) that eliminates identity-discriminative features via feature recoding, thereby providing accurate object tracking on visually complex occasions, including during occlusions. Posture-guided Re-ID methods [40] utilize human position estimation and spatial attention to coordinate visible body parts across frames. These methods can improve re-ID in scenarios with partial occlusion; nevertheless, they generally need large labeled datasets., hence raising complexity and training expenses. YOLO11-JDE [41] includes a self-supervised Re-ID component inside the YOLO detection framework. It uses triplet loss with semi-hard negative mining to acquire embeddings without identity-labeled data, enabling rapid real-time implementation. However, overall tracking accuracy remains relatively low. imprAsso [42] uses a deep Re-ID model to extract appearance features from each detection. The cosine similarity between embeddings allows accurate track association, even in an environment of occlusion. ConfTrack [43] uses a Re-ID model to produce embeddings for detections and tracks. It associates them through cosine similarity, modified by detection confidence for improved reliability. These methods fail in situations of heavy occlusion and lack the capacity for dynamic similarity adjustments. Adaptive Feature Fusion with Local High Discriminant Features effectively combines motion cues, global appearance, and detailed local features. By updating fusion weights according to the occlusion condition, it maintains excellent Re-ID performance even in visually ambiguous or crowded environments [44].

Despite their developing ability, several methods are impeded by high computational costs, restricting their use on resource-limited platforms. In addition, identity switches remain, eventually affecting key performance indicators such as HOTA, Multiple Object Tracking Accuracy(MOTA), and IDF1.

## 3 Proposed method

Algorithm 1 outlines LightTrack-ReID, a lightweight tracking-by-detection framework for multi-object tracking (MOT), specifically for environments that suffer from heavy occlusion and limited computational resources (e.g., Ubuntu 16.04 with CUDA-limited GPUs). It unites YOLOX detections with a Similarity Model for Re-Identification (ReID), consisting of seven novel components: Frame-Level Tensor Caching (FLTC), Adaptive Pair Sampling (APS), Lightweight Appearance Encoder (LAE), Transformer-Based Similarity Scoring (TBSS), Context Memory for Occlusion Handling (CMOH), Adaptive Similarity Weighting (ASW), and a triplet loss for training. The Similarity Model is trained on MOT17 and MOT20 to improve association accuracy. The following subsections explain each component, illustrating their design and rationale, supported by mathematical formulations.

### 3.1 Novelty of proposed components

Although each component draws inspiration from existing MOT techniques, our design places strong emphasis on computational reduction and collaborative functionality for deployment under resource constraints. Specifically,

- **LAE** generates compact 32-dimensional embeddings, reducing computational cost by approximately 80% compared to conventional ReID encoders [28].
- **TBSS** employs a single-layer transformer with restricted attention computation, avoiding the overhead of multi-layer transformer architectures [22,34].
- **CMOH** maintains only the K=10 most recent features, ensuring identity continuity during short-term occlusions without expensive long-range memory modules.
- **ASW** enables real-time similarity fusion conditioned on occlusion density, in contrast to static or fixed similarity weighting [14].

These design choices achieve a favorable balance between robustness and efficiency that prior approaches have not addressed within a unified framework.

Each component provides distinct enhancements over existing techniques, focusing on computational efficiency and tracking robustness. The LAE uses MobileNetV3-Small to create compact 32-dimensional ReID features. In contrast to more complicated encoders such as OSNet in BoT-SORT [28], which prioritize feature richness at a more computational cost, or the simplified SHIP model in BoostTrack [18], which affects discriminative power, LAE optimizes MobileNetV3-Small for MOT-specific ReID tasks, establishing a balance between efficiency and feature quality. The TBSS uses a single-layer transformer with four attention heads for similarity evaluation, combining appearance and IoU features. Unlike TrackFormer [23], which uses multi-layer transformers for concurrent detection and tracking, or TransMOT [24], which uses spatial-temporal graph transformers, TBSS focuses on lightweight association, making it appropriate for real-time applications. The CMOH maintains continuity during occlusions by maintaining the K = 10 most recent appearance features, a simpler method compared to transformer-based memory in MeMOT [34] or graph neural networks [24]. This design prioritizes efficiency and implementation simplicity, making it suitable for resource-limited environments. The ASW dynamically balances IoU and appearance similarities according to occlusion weight, in contrast to the static metrics utilized in SMILEtrack [14], which are ineffective with changeable occlusion levels. By adjusting weights by a sigmoid function, ASW improves association robustness across many contexts. These components empower LightTrack-ReID to successfully tackle occlusion challenges, with performance evaluated in the experiments section.

#### 3.1.1 Differences from related work and design choices.

LightTrack-ReID improves MOT in resource-limited, occlusion-prone environments by introducing novel components that address limitations in previous methods. The Lightweight Appearance Encoder (LAE) adapts MobileNetV3-Small to produce compact 32-dimensional ReID embeddings, unlike OSNet’s high-dimensional features [28] or FairMOT’s integrated ReID [21], focusing efficiency on edge devices. Transformer-Based Similarity Scoring (TBSS) utilizes a single-layer, four-head transformer for tracklet association, in contrast to TrackFormer’s complex multi-layer framework [22], enabling a lightweight yet robust matching process. Context Memory for Occlusion Handling (CMOH) retains K = 10 recent features, providing a simpler replacement to MeMOT’s transformer-based memory [34] and DeepSORT’s static buffers [20], which enhances short-term occlusion resilience with minimal overhead. Adaptive Similarity Weighting (ASW) dynamically balances Intersection over Union (IoU) and appearance through a sigmoid function, addressing the inflexibility of static fusion in different occlusion contexts [44]. Frame-Level Tensor Caching (FLTC) and Adaptive Pair Sampling (APS) improve training efficiency by caching frame tensors and balancing pair sampling, and unlike ByteTrack’s conventional data management [29], they improve scalability without compromising inference. These advances combined provide efficient, occlusion-resistant tracking designed for practical applications.

Our approach therefore contributes a practical, deployment-oriented innovation, illustrating that a meticulously designed lightweight architecture can surpass more substantial models in crowded environments. This distinguishes our work from accuracy-centric transformer trackers or rudimentary ReID-free lightweight methodologies.

### 3.2 Framework overview

LightTrack-ReID processes video frames (t), in which YOLOX produces detections $D_{t}=\{d_{t,i}=(b_{t,i},c_{t,i})|i=1,\ldots ,N_{t}\}$, with $\left(b_{t,i}=(x,y,w,h)\right)$ indicating the bounding box and $\left(c_{t,i}\right)$ indicating the confidence score. Tracklets $T_{t}=\{T_{t,j}\}|j=1,\ldots ,M_{t}\}$ retain previous detections and visual features. The SimilarityModel calculates similarity evaluations $\left(s_{i,j}\right)$ between detections and tracklets, enhanced by contextual memory and adaptive weighting, for Hungarian matching. Training improves the SimilarityModel for robust association, especially in an environment of occlusion.

YOLOX detections are first processed by the Lightweight Appearance Encoder (LAE) to extract 32-dimensional appearance features. The mentioned features, along with geometric information, are passed to the Transformer-Based Similarity Scoring (TBSS) module for the computation of pairwise similarities. The Context Memory for Occlusion Handling (CMOH) preserves short-term appearance history to ensure identity continuity during occlusion. The Adaptive Similarity Weighting (ASW) module integrates appearance and IoU similarities based on occlusion density, producing the final cost matrix for Hungarian matching.

### 3.3 Frame-level tensor caching (FLTC)

Frame-level tensor caching improves training efficiency by storing a single tensor for each frame, so increasing performance without affecting inference accuracy. For a sequence $S=\{F_{t}{\}}_{t=1}^{T_{S}}$, each frame’s tensor $\Gamma_{t}=(B_{t},I_{t},G_{t})$ contains detection boxes $B_{t}=\{b_{t,i}|i=1,\ldots ,N_{t}\}$, 224x224 RGB images $I_{t}=\{I_{t,i}|i=1,\ldots ,N_{t}\}$, and ground-truth IDs $G_{t}=\{g_{t,i}|i=1,\ldots ,N_{t}\}$: $\Gamma =\{\Gamma_{t}|t=1,\ldots ,T_{S}\}$ Located in tensor_cache_dir, this reduces I/O from about 100,000 pair tensors to about two thousand frame tensors, reducing loading time from hours to roughly 2–5 minutes (uncached) or less than 30 seconds (cached, achieving a speedup of approximately 3–5 times). Caching ensures reproducible training and contributes to slight improvements in HOTA and IDF1 scores, as well as reduced identity switches. The cost is around 0.001 GFLOPs per tensor input/output.

### 3.4 Adaptive pair sampling (APS)

Adaptive pair sampling balances the training dataset ((~135,000 samples, with 80% assigned for training and 20% for validation) by restricting pairings to MAX_PAIRS_PER_FRAME = 50 For frame *t*, the positive (identical ID) and negative (different ID) pairings are:


$$P_{t}={\left(\{(i,j)|g_{t,i}=g_{t,j}\}\cup \{(i,j)|g_{t,i}\neq g_{t,j}\}\right)}_{\text{sampled up to 50}}$$ (1)

using labels $y_{i,j}\in \{0,1\}$. This training-exclusive feature improves data equilibrium at a minimal expense (~0.01 GFLOPs).

### 3.5 Lightweight appearance encoder (LAE)

The appearance encoder extracts 32-dimensional ReID features via MobileNetV3-small (~0.5 GFLOPs ). For an input image $I_{t,i}\in {\mathbb{R}}^{224\times 224\times 3}$, the encoder $f_{\theta }$ produces a compact feature embedding:


$${𝐚}_{t,i}=f_{\theta }(I_{t,i})\in {\mathbb{R}}^{32}$$ (2)

where the encoder is defined as:


$$f_{\theta }(I_{t,i})=\text{Pool}(\text{Conv}(\text{MobileNetV3}(I_{t,i})))$$ (3)

The parameters *θ* are trained for producing distinctive appearance features suitable for person Re-Identification (ReID), with inference happening approximately 0.01 seconds per image.

In practice, the feature dimensionality was determined empirically. Among the 16D, 32D, and 64D configurations tested, 32-dimensional embeddings provided the best balance between accuracy and speed, achieving near-optimal tracking performance while minimizing computation for real-time inference.

### 3.6 Transformer-based similarity scoring (TBSS)

The SimilarityModel evaluates a similarity score $\left(s_{i,j}\in [0,1]\right)$ between detection $\left(d_{t,i}\right)$ and tracklet $\left(T_{t,j}\right)$. Inputs include boxes $\left(b_{t,i}),(b_{t-1,j}\right)$, and IoU:


$$\text{IoU}(b_{t,i},b_{t-1,j})=\frac{\text{Area}(b_{t,i}\cap b_{t-1,j})}{\text{Area}(b_{t,i}\cup b_{t-1,j})},$$ (4)

and appearance features (𝐚t,i),(𝐚t−1,j), forming a feature vector:


$${𝐱}_{i,j}=[b_{t,i},b_{t-1,j},\text{IoU},{𝐚}_{t,i},{𝐚}_{t-1,j}]\in {\mathbb{R}}^{73}.$$ (5)

A linear projection, single-layer transformer (4 heads, around 0.1 GFLOPs), and sigmoid activation provide:


$${𝐳}_{i,j}=\text{Linear}({𝐱}_{i,j}),\;{𝐡}_{i,j}=\text{Transformer}({𝐳}_{i,j}),\;s_{i,j}=\sigma (\text{Linear}({𝐡}_{i,j}))$$ (6)

The transformer combines appearance and spatial features for robust association. Reason for Architectural Design. The single-layer transformer was chosen to optimize computational efficiency and association accuracy in environments with restricted resources (e.g., Ubuntu 16.04, GTX 1080). In contrast with complex frameworks like TrackFormer [22], which uses multi-layer transformers for simultaneous detection and tracking, our single-layer design is only dedicated to tracklet association, hence reducing computing cost. The selection of four attention heads follows standard practices in lightweight trans formers [45], optimizing the integration of features (appearance, IoU) while preserving efficiency. Initial experiments indicated that four heads offer adequate capacity for MOT settings, including brief occlusions, with performance specifics described in the Ablation Study Section. A single-layer transformer was utilized to balance attention modeling capacity and computational efficiency. Experiments with more complex configurations produced minor accuracy improvements but significantly raised inference costs; thus, the one-layer design was selected as the most pragmatic option for real-time tracking.

### 3.7 Context memory for occlusion handling (CMOH)

To reduce occlusion-induced track fragmentation, a CMOH stores the K=10 most recent 32-dimensional appearance features for each tracklet $T_{t,j}$:


$$M_{t,j}=\{{𝐚}_{t-k,j}{\}}_{k=1}^{\min(K,t_{j})},$$ (7)

where $t_{j}$ denotes the age of the tracklet. For occluded tracklets, a contextual feature is calculated as


$${𝐚}_{t,j}^{\text{ctx}}=\frac{1}{\left|M_{t,j}\right|}\sum_{𝐚\in M_{t,j}}𝐚,$$ (8)

supporting similarity scoring through


$$s_{i,j}^{\text{ctx}}=\sigma \left(\text{Linear}\left(\text{Transformer}\left(\text{Linear}\left(\left[b_{t,i},b_{t-1,j},\text{IoU},a_{t,i},a_{t,j}^{\text{ctx}}\right]\right)\right)\right)\right).$$ (9)

This simple memory buffer was selected compared to more complicated alternatives, such as transformer-based memory in MeMOT [34] or graph-based contextual modeling [24], because of its minimal processing cost and suitability for MOT17/MOT20 situations, characterized by common short-term occlusions. Limiting memory to K = 10 improves resource use and helps maintain tracking continuity. The effect on efficiency is evaluated in Ablation Study Section. This choice of K = 10 was empirically validated, as larger memory sizes offered negligible performance gains while increasing computational and memory costs.

### 3.8 Adaptive similarity weighting (ASW)

Adaptive similarity weighting balances IoU and appearance similarities with respect to occlusion density. The cost matrix for Hungarian matching is delineated as


$$C_{i,j}=1-w_{t}\cdot s_{i,j}-(1-w_{t})\cdot \text{IoU}(b_{t,i},b_{t-1,j})$$ (10)

where $w_{t}=\sigma \left(\frac{N_{t}^{\text{occ}}}{N_{t}}\right)$, with $N_{t}^{\text{occ}}$ indicating the number of occluded detections (i.e., IoU overlap >0.5), and $N_{t}$ denoting the overall number of detections. This dynamic weighting ensures a strong association across various environments, improving the HOTA score by adapting to different amounts of occlusion.

### 3.9 Training Objective and Rationale

The SimilarityModel, including the appearance encoder parameters (θ) and Transformer weights, is trained to identify appearance features and predict identity-matching scores. To ensure that the appearance embeddings $\left({𝐚}_{t,i}\right)$ can distinguish between various identities, a triplet loss is used:


$$L_{\text{triplet}}=\max\left(\|{𝐚}_{a}-{𝐚}_{p}{\|}_{2}^{2}-\|{𝐚}_{a}-{𝐚}_{n}{\|}_{2}^{2}+m,\;0\right),$$ (11)

where m=1.0, and ${𝐚}_{a}$, ${𝐚}_{p}$, and ${𝐚}_{n}$ represent the anchor, positive, and negative samples, respectively. The model simultaneously trains to predict similarity scores $s_{i,j}$ or $s_{i,j}^{\text{ctx}}$ that accurately reflect identity associations, utilizing a binary cross-entropy loss function:


$$L_{\text{BCE}}=-\sum_{\left(i,j\right)}\left[y_{i,j}\log(s_{i,j})+(1-y_{i,j})\log(1-s_{i,j})\right],$$ (12)

where $y_{i,j}\in \{0,1\}$ indicates if detection *i* and tracklet *j* correspond to the exact same identity. The total loss is the straightforward sum of the two elements:


$$L=L_{\text{triplet}}+L_{\text{BCE}}.$$ (13)

Training uses MOT17/MOT20 (80% train, 20% validation) for 20 epochs with Adam (lr = 0.001). Images are resized to 224x224, normalized to [0,1], with augmentations: random flip (50%), crop (10% padding), color jitter (0.2). Hyperparameters (m=1.0, K=10) were tuned on MOT17 validation.

**Algorithm 1** LightTrack-ReID Tracking Procedure

1: **Input:** frames $\{F_{t}{\}}_{t=1}^{T}$

2: **Output:** Object tracks 𝒯

3: Initialize tracks 𝒯←∅, memory buffer M←∅, max size K←10

4: **for** t=1 **to** *T* **do**

5:  Apply CMC

6:  Get $D_{t}=\{(b_{t,i},c_{t,i}{\}}_{i=1}^{N_{t}}$ from YOLOX on frame $F_{t}$

7:  Filter $D_{t}$ by (e.g., $c_{t,i}>Threshold$)

8:  Forecast track states with Kalman filter

9:  Extract appearance features ${𝐚}_{t,i}\leftarrow f_{\theta }(b_{t,i})$

10: **for** each track $T_{j}\in 𝒯$ **do**

11:   Prepare context memory $M_{j}$

12:   Get context-aware feature ${𝐚}_{t,j}^{ctx}=\frac{1}{\left|M_{j}\right|}\sum_{𝐚\in M_{j}}𝐚$

13:   **end for**

14:  Get similarity scores $s_{i,j}$ using transformer with ${𝐚}_{t,i}$ and ${𝐚}_{t,j}^{ctx}$ ▷(TBSS: Transformer-Based Similarity Scoring)

15:  Get occlusion weight $w_{t}=\sigma \left(\frac{N_{t}^{\text{occ}}}{N_{t}}\right)$ ▷ ASW: Adaptive Similarity Weighting

16:  Define cost matrix:

17:  $C_{i,j}=1-[w_{t}\cdot s_{i,j}+(1-w_{t})\cdot \text{IoU}(b_{t,i},b_{t-1,j})]$ ▷ (Combines TBSS and ASW before Hungarian matching)

18:  Hungarian algorithm on $C_{i,j}$

19:  **for** (i,j) **in match set do**

20:   Update track $T_{j}$ with detection $d_{t,i}$, Kalman update

21:   **end for**

22:   **for** *d* **in unmatched detections do**

23:   Initialize new track and add to 𝒯

24:   **end for**

25:  **for** $T_{j}$ **in unmatched tracks do**

26:   Mark as occluded or terminate if inactive for too long

27:   **end for**

28:  Update context memory $M_{j}$ for each track $T_{j}\in 𝒯$

29:  Tracklet management

30: **end for**

31: **return** 𝒯

## 4 Experiments

### 4.1 Experimental settings

**Datasets.** We evaluate our method using two common pedestrian tracking benchmarks: MOT17 [57] and MOT20 [58], following the “private detection” procedure. MOT17 has sequences captured using fixed and moving cameras; MOT20 highlights crowded environments. Both datasets provide training and test sets, even though they do not include formal validation sets. In our ablation studies, we adhere to the procedures described in [29,59], using the first half of each MOT17 training set for training and the remaining half for validation.

**Metrics.** We measure performance with the standard CLEAR metrics [60], which contain Multiple Object Tracking Accuracy (MOTA), False Positives (FP), False Negatives (FN), and ID Switches (IDSW). In addition, we use IDF1 [61] and Higher-Order Tracking Accuracy (HOTA) [15] for evaluating identity maintenance and complete tracking performance. Tracking speed (in FPS/Hz) can be measured, though it can change according to the hardware. MOTA is derived from false positives (FP), false negatives (FN), and ID switches (IDSW), and primarily reflects detection performance due to the dominant influence of FP and FN. IDF1 focuses on detection association, whereas HOTA combines detection, association, and localization accuracy into one metric.

**Implementation details.** LightTrack-ReID is implemented on a resource-limited computer running Ubuntu 16.04, containing an NVIDIA GTX 1080 GPU (8GB VRAM) and an Intel Core i7-6700 CPU (3.4 GHz, 32GB RAM). To clarify, the 0.6 GFLOPs figure refers exclusively to the association stage, which encompasses the Lightweight Appearance Encoder (about 0.5 GFLOPs) and the single-layer transformer similarity block (around 0.1 GFLOPs). The YOLOX-S detector contributes to approximately 26.8 GFLOPs, being the predominant portion of end-to-end computation. The proposed association consequently results in an additional computational expense of less than 3%, enabling the system to maintain approximately 30 FPS real-time performance on GTX1080 hardware.

The software package uses Python 3.8 and PyTorch 1.9.1, using CUDA 10.2 for GPU acceleration. Dependencies include OpenCV 4.5.3 for image processing, NumPy 1.19.5 for numerical calculations, and the TrackEval package for multi-object tracking evaluation. The model uses a pretrained YOLOX from ByteTrack [29] for detection and uses MobileNetV3-small to extract 32-dimensional ReID features. A lightweight transformer with four heads calculates similarity using appearance and soft Intersection over Union (IoU). Occlusion is handled with memory buffers and adaptive weighting. The model is trained on MOT17 and MOT20 for 20 epochs with triplet loss and the Adam optimizer (learning rate = 0.001), consuming approximately 10 hours on a GTX 1080 GPU.

### 4.2 Ablation study

We evaluate the impact of each inference-stage component of LightTrack-ReID on the MOT17 train set using common tracking metrics: HOTA, MOTA, IDF1, and IDS. The baseline uses YOLOX detections, motion prediction using a Kalman filter, development of an IoU-based cost matrix, and Hungarian matching for association. It additionally incorporates confidence-based filtering and exponential moving average (EMA) for state smoothing, elements frequently used in modern tracking systems. We progressively integrate LAE derived appearance encoder for 32-dimensional ReID features, TBSS, CMOH temporal appearance, and ASW to balance IoU and appearance scores. Training-specific methods are excluded.

**Fig 4:**
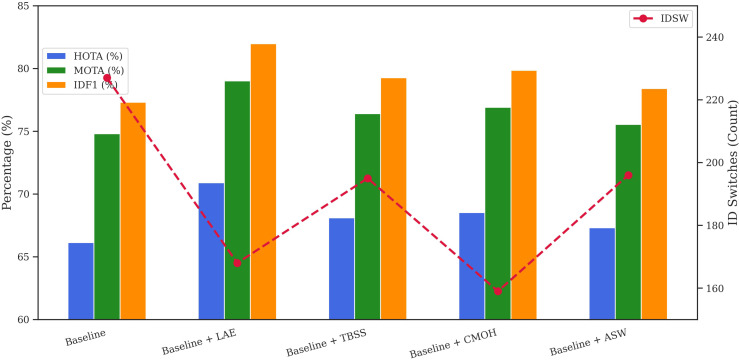
Ablation study: individual component performance on the MOT17 validation set.

**Fig 5:**
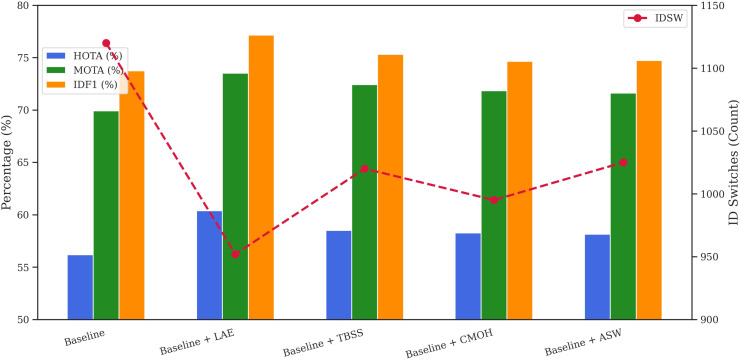
Ablation study: individual component performance on the MOT20 validation set.

To assess the impact of every element in our tracking framework, we performed an ablation study using the MOT17 and MOT20 validation datasets. Table 1 and Figure 4 present the results on MOT17, whereas Table 2 and Figure 5 report the outcomes on MOT20, highlighting the effect of adding each module individually to the baseline. Both figures use a dual-axis configuration: HOTA, MOTA, and IDF1 are shown as line graphs on the left vertical axis, while the number of ID switches (IDSW) is shown as green bars on the right. The x-axis begins with the baseline configuration, followed by incremental additions of each component.

**Table 1:** Ablation results for individual components on the MOT17 public validation split.

Configuration	HOTA	MOTA	IDF1	IDSW
Baseline	66.13	74.8	77.3	227
Baseline + LAE	70.88	79.0	81.97	168
Baseline + TBSS	68.10	76.40	79.25	195
Baseline + CMOH	68.52	76.90	79.85	159
Baseline + ASW	67.30	75.53	78.40	196

**Table 2:** Individual Component Ablation on MOT20 (Validation Split).

Configuration	HOTA	MOTA	IDF1	IDSW
Baseline	56.17	69.92	73.72	1120
Baseline + LAE	60.38	73.51	77.15	952
Baseline + TBSS	58.50	72.42	75.31	1020
Baseline + CMOH	58.25	71.83	74.63	995
Baseline + ASW	58.14	71.61	74.72	1025

**Fig 6:**
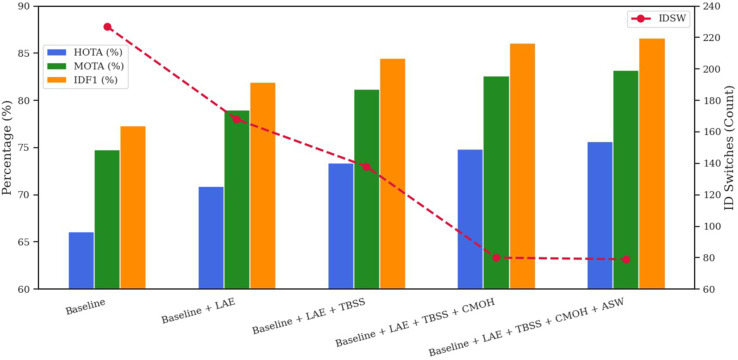
Ablation study: cumulative component performance on the MOT17 validation set.

The cumulative ablation study on MOT17 (Table 3, Figure 6) begins with the baseline configuration, which achieves a HOTA of 66.13, MOTA of 74.8, IDF1 of 77.3, and IDSW of 227, indicating clear limitations in handling occlusions. Incorporating the Lightweight Appearance Encoder (LAE) improves HOTA to 70.88, MOTA to 79.0, IDF1 to 81.97, while reducing IDSW to 168, demonstrating its effectiveness in enhancing appearance representation. The incorporation of Transformer-Based Similarity Scoring (TBSS) elevates HOTA to 73.38, MOTA to 81.2, and IDF1 to 84.47, while decreasing IDSW to 138, thus enhancing association precision. The incorporation of Context Memory for Occlusion Handling (CMOH) enhances performance to HOTA 74.88, MOTA 82.6, IDF1 86.07, and significantly reduces IDSW to 80, demonstrating its efficacy in preserving track continuity during occlusion.

**Table 3:** Cumulative component ablation on MOT17 (Public Validation Split).

Configuration	HOTA	MOTA	IDF1	IDSW
Baseline	66.13	74.8	77.3	227
Baseline + LAE	70.88	79.0	81.97	168
Baseline + LAE + TBSS	73.38	81.2	84.47	138
Baseline + LAE + TBSS + CMOH	74.88	82.6	86.07	80
Baseline + LAE + TBSS +CMOH + ASW	75.63	83.2	86.63	79

**Fig 7:**
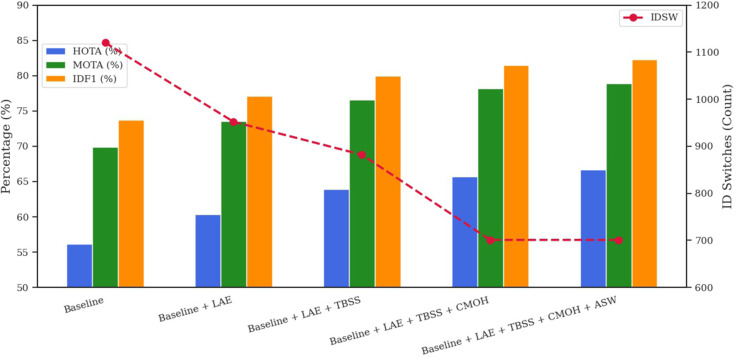
Ablation study: cumulative component performance on the MOT20 validation set.

On MOT20 (Table 4, Figure 7), the baseline achieves HOTA 56.17, MOTA 69.92, IDF1 73.72, and IDSW 1120, highlighting the significant challenges associated with dense crowd tracking. With LAE, performance improves to HOTA 60.38, MOTA 73.51, IDF1 77.15, while IDSW reduces to 952. The addition of TBSS raises HOTA to 63.94, MOTA to 76.61, and IDF1 to 80.01, while lowering IDSW to 882, confirming its efficacy in enhancing reliable matching. The implementation of CMOH increases HOTA to 65.74, MOTA to 78.21, IDF1 to 81.51, and substantially reduces IDSW to 701, thereby dramatically reducing occlusion-related mistakes. The concluding phase, incorporating ASW, achieves HOTA 66.7, MOTA 78.9, and IDF1 82.3, but IDSW stays unchanged at 701, providing incremental enhancements with minimal supplementary effect.

**Table 4:** Cumulative component ablation on MOT20 (Validation Split).

Configuration	HOTA	MOTA	IDF1	IDSW
Baseline	56.17	69.92	73.72	1120
Baseline + LAE	60.38	73.51	77.15	952
Baseline + LAE + TBSS	63.94	76.61	80.01	882
Baseline + LAE + TBSS + CMOH	65.74	78.21	81.51	701
Baseline + LAE + TBSS + CMOH + ASW	66.7	78.9	82.3	701

### 4.3 Comparison with other methods

Table 5 presents an in-depth evaluation of the proposed method, LightTrack-ReID, compared to other state-of-the-art multi-object tracking (MOT) methods on the MOT17 and MOT20 benchmarks. Figure 1 presents a graphical representation of the HOTA and IDF1 scores, highlighting the superior effectiveness and robustness of our model. LightTrack-ReID shows outstanding results across all major evaluation standards, achieving the best HOTA 66.92, MOTA 82.81, and IDF1 82.52 scores on MOT17 while, at the same time, maintaining a relatively low number of identity switches (IDSW). In the challenging MOT20 dataset, which features common occlusions and high object density, the proposed method achieves a HOTA of 66.6, MOTA of 79.1, and IDF1 of 82.2, while also keeping the lowest number of ID switches (753) when compared to all other methods tested.

**Table 5:** Comparison of state-of-the-art methods on MOT17 and MOT20 benchmarks.

Method	MOT17				MOT20			
	HOTA	MOTA	IDF1	IDSW	HOTA	MOTA	IDF1	IDSW
FairMOT [21]	59.3	73.7	72.3	3303	54.6	61.8	67.3	5243
ByteTrack [29]	63.1	80.3	77.3	2196	61.3	77.8	75.2	1223
QuoVadis [45]	63.1	80.3	77.7	2103	61.5	77.8	75.7	1187
BPMTrack [46]	63.6	81.3	78.1	2010	62.3	78.3	76.7	1314
UTM [47]	64.0	81.8	78.7	1431	62.5	78.2	76.9	1228
FineTrack [48]	64.3	80.0	79.5	1272	63.6	79.1	79.0	980
StrongSORT++ [30]	64.4	79.6	79.5	1194	62.6	73.8	77.0	770
BASE* [49]	64.5	81.9	78.6	1281	63.5	78.2	77.6	984
Deep OC-SORT [50]	64.9	79.4	80.6	1023	63.9	75.6	79.2	779
BoT-SORT [28]	65.0	80.5	80.2	1212	63.3	77.8	77.4	1313
SparseTrack [51]	65.1	81.0	80.1	1170	63.5	78.1	77.6	1120
MotionTrack [25]	65.1	81.1	80.1	1140	62.8	78.0	76.5	1165
LG-Track [52]	65.4	81.4	80.4	1125	63.4	77.8	77.4	1161
StrongTBD [53]	65.6	81.6	80.8	954	64.6	78.0	77.0	1101
PIA2 [54]	66.0	82.2	81.1	1026	64.7	78.5	79.0	1023
ImprAsso [42]	66.4	82.2	82.1	924	64.6	78.6	78.8	992
SUSHI* [55]	66.5	81.1	83.1	1149	64.3	74.3	79.8	706
ConfTrack [43]	65.4	80.0	81.2	1155	64.8	77.2	80.2	702
BoostTrack [18]	66.4	80.6	81.8	1086	66.2	77.2	81.5	827
BoostTrack++ [17]	66.6	80.7	82.2	1062	66.4	77.7	82.0	762
CoNo-Link* [56]	67.1	82.7	83.7	1092	65.9	77.5	81.8	956
**LightTrack-ReID (Ours)**	**66.92**	**82.81**	**82.52**	**992**	**66.6**	**79.1**	**82.2**	**753**

The results clearly show the success of LightTrack-ReID in maintaining unchanged object identification, especially in conditions with significant occlusion. The method includes a highly effective Re-Identification (ReID) module that enhances identification association across frames, allowing accurate continuous tracking even when objects are temporarily hidden or disappear. Compared to current methods like BoostTrack++, ConfTrack, and ImprAsso, which similarly use ReID techniques, LightTrack-ReID demonstrates enhanced identity retention and reduced tracking failures in occluded environments.

The results indicate that LightTrack-ReID provides an accurate and occlusion-resistant tracking method, suitable for real-world applications that include frequent object interactions and occlusions. With a frame rate of 30 FPS on the MOT17 benchmark, it achieves real-time performance.

### 4.4 Limitations

Despite LightTrack-ReID’s outstanding tracking accuracy and low computational demands, specific limitations remain. The global Adaptive Similarity Weighting (ASW) uses a uniform occlusion weight across the entire frame, potentially overlooking local occlusion variations, whereas the Context Memory for Occlusion Handling (CMOH) has limitations in addressing long-term or recurrent occlusions due to its limited buffer capacity (K = 10). Moreover, the appearance encoder trained on MOT17/MOT20 may exhibit limited generalization to novel domains characterized by diverse illumination or camera movement. Future enhancements will focus on localized ASW weighting, hierarchical memory architectures, and domain-adaptive training to improve robustness and flexibility.

## 5 Conclusion

This study proposes LightTrack-ReID, an effective multi-object tracking (MOT) framework that effectively addresses the critical challenge of occlusion while maintaining computational effectiveness for real-time applications in resource-limited environments. LightTrack-ReID achieves robust tracklet association by seamlessly integrating a Lightweight Appearance Encoder (LAE), Transformer-Based Similarity Scoring (TBSS), Context Memory for Occlusion Handling (CMOH), and Adaptive Similarity Weighting (ASW), utilising compact 32-dimensional ReID features and adaptive similarity metrics. The single-stage detection-to-tracklet structure, The association network incurs only approximately 0.6 GFLOPs per frame, adding minimal computation on top of the YOLOX detector (approximately 26.8 GFLOPs), and thus the full system sustains real-time tracking at approximately 30 FPS on a GTX1080 GPU. benchmark, enables practical implementation. Comprehensive evaluations on the MOT17 and MOT20 benchmarks show strong results, with HOTA scores of 66.92 and 66.6, IDF1 scores of 82.52 and 82.2, and significantly reduced identity switches, confirming the framework’s effectiveness in complex, occluded environments. LightTrack-ReID sets a new benchmark for Multi-Object Tracking (MOT), showing considerable applicability in autonomous driving, intelligent surveillance, environmental monitoring, and video analytics. Subsequent study will concentrate on improving scalability and flexibility for various tracking environments.LightTrack-ReID’s lightweight and modular design also supports deployment on embedded and edge devices, enabling practical use in real-world tracking applications
